# *ADAM10* Gene Variants in AD Patients and Their Relationship to CSF Protein Levels

**DOI:** 10.3390/ijms24076113

**Published:** 2023-03-24

**Authors:** Pablo Agüero-Rabes, Julián Pérez-Pérez, Lucía Cremades-Jimeno, María-Salud García-Ayllón, Adriana Gea-González, María José Sainz, Ignacio Mahillo-Fernández, Raquel Téllez, Blanca Cárdaba, Javier Sáez-Valero, Estrella Gómez-Tortosa

**Affiliations:** 1 Department of Neurology, Fundación Jiménez Díaz, 28040 Madrid, Spain; pablo.aguero@quironsalud.es (P.A.-R.); mjsainz@fjd.es (M.J.S.); 2 Secugen S.L., 28040 Madrid, Spain; j.perez@secugen.es; 3 Department of Immunology, IIS-Fundación Jiménez Díaz-UAM, 28040 Madrid, Spain; lucia.cremades@quironsalud.es (L.C.-J.); raquel.tellez@quironsalud.es (R.T.); bcardaba@quironsalud.es (B.C.); 4 Instituto de Neurociencias de Alicante, Universidad Miguel Hernández-CSIC, 03550 Alicante, Spain; ms.garcia@umh.es (M.-S.G.-A.); agea@umh.es (A.G.-G.); j.saez@umh.es (J.S.-V.); 5 Centro de Investigación Biomédica en Red sobre Enfermedades Neurodegenerativas (CIBERNED), 03550 Alicante, Spain; 6 Unidad de Investigación, Hospital General Universitario de Elche, Fundación para el Fomento de la Investigación Sanitaria y Biomédica de la Comunitat Valenciana (FISABIO), 46020 Valencia, Spain; 7 Department of Epidemiology and Biostatistics, Fundación Jiménez Díaz, 28040 Madrid, Spain; imahillo@fjd.es; 8 Centro de Investigación Biomédica en Red Sobre Enfermedades Respiratorias (CIBERES), 28029 Madrid, Spain; 9 Instituto de Investigación Sanitaria y Biomédica de Alicante (ISABIAL), 03010 Alicante, Spain

**Keywords:** Alzheimer’s disease, ADAM10, α-secretase, disintegrin metalloproteinase 10 protein, genetics, CSF biomarkers

## Abstract

ADAM10 is the main α-secretase acting in the non-amyloidogenic processing of APP. We hypothesized that certain rare *ADAM10* variants could increase the risk for AD by conferring the age-related downregulation of α-secretase. The *ADAM10* gene was sequenced in 103 AD cases (82% familial) and 96 cognitively preserved nonagenarians. We examined rare variants (MAF < 0.01) and determined their potential association in the AD group with lower CSF protein levels, as analyzed by means of ELISA, and Western blot (species of 50 kDa, 55 kDa, and 80 kDa). Rare variants were found in 15.5% of AD cases (23% early-onset, 8% late-onset) and in 12.5% of nonagenarians, and some were group-specific. All were intronic variants except Q170H, found in three AD cases and one nonagenarian. The 3′UTR rs74016945 (MAF = 0.01) was found in 6% of the nonagenarians (OR 0.146, *p* = 0.057). Altogether, ADAM10 total levels or specific species were not significantly different when comparing AD with controls or carriers of rare variants versus non-carriers (except a Q170H carrier exhibiting low levels of all species), and did not differ according to the age at onset or *APOE* genotype. We conclude that *ADAM10* exonic variants are uncommon in AD cases, and the presence of rare intronic variants (more frequent in early-onset cases) is not associated with decreased protein levels in CSF.

## 1. Introduction

All known genetic causes of Alzheimer’s disease (AD) are related to the abnormal processing of the amyloid precursor protein (APP) and subsequent pathological accumulation of Aβ peptides in the brain [1]. In the non-amyloidogenic processing of APP, the protein is proteolyzed through the α-secretase pathway, resulting in soluble fragments (sAPPα) formation and precluding the further generation of Aβ peptides [2]. Several enzymes of the disintegrin and metalloprotease (ADAM) family have α-secretase activity in vitro. Particularly, ADAM10 has been identified as the major α-secretase responsible for the ectodomain shedding of APP in the brain [3,4,5].

Both cell and animal models have demonstrated that the full activity of ADAM10 is essential to prevent β-amyloid deposits [5,6,7,8]. The over-expression of ADAM10 in mouse models has been shown to halt Aβ production and subsequent aggregation [8], while the downregulation of ADAM10—through increased levels of its inhibitor, SFRP1 protein—anticipated the appearance of Alzheimer changes in an AD-like mouse model [9]. Moreover, a non-transgenic mouse model that mimics the early stages of AD was obtained by blocking ADAM10 activity (together with its synapse-associated protein partner), which shifted APP metabolism towards amyloidogenesis [10]. In addition to APP proteolysis, ADAM10 is known to cleave a number of other proteins, and also plays a role in synaptic functions, in the regulation of dendritic spine formation and stabilization, and in regulating the astrocytic immune response [11,12].

Several clinical studies have described decreased α-secretase activity in sporadic AD cases compared to controls based on analyses of ADAM10 or its neurotrophic metabolite, sAPPα. There is evidence of an overall decrease of ADAM10 mRNA in brain tissue; a protein decrease in platelets, plasma exosomes, cerebrospinal fluid (CSF); as well as a sAPPα decrease in platelets and CSF [13,14,15,16,17]. In contrast, ADAM10 levels are increased in the platelets of cognitively preserved octogenarians, suggesting that enhanced α-secretase activity contributes to cognitively healthy aging or confers resilience to neurodegeneration [18]. However, a more recent study supports that ADAM10 levels are increased in plasma and CSF of cases with mild AD, though in these samples the protease was shown to be inactive [19]. As ADAM10 is active as a membrane-anchored protease, it is possible that soluble ADAM10 that has undergone ectodomain shedding represents a soluble inactive protein, and increased levels could be linked to reduced overall enzyme activity [11,19,20]

On the genetic level, *ADAM10* is considered a top candidate gene for involvement in the pathophysiology of AD due to the anti-amyloidogenic role of the protein [21]. Some *ADAM10* variants have been associated with a higher susceptibility to late-onset AD. Kim et al. [22] reported two rare missense mutations (Q170H, R181G) in late-onset AD kindreds. Co-segregation of the variants with the disease was incomplete in these families, but the mutations were shown to promote AD-type changes in transgenic mice [23]. Suh et al. [23] showed that both variants impair the molecular chaperone function of the ADAM10 prodomain, attenuate the α-secretase activity of the protein, and shift APP processing towards β-secretase-mediated cleavage, thereby enhancing Aβ plaque load. The presence of the mutations also reduced the effect of ADAM10 as a stimulator of adult hippocampal neurogenesis. In addition, we recently reported an AD family carrying a heterozygous nonsense mutation in *ADAM10* (p.Tyr167*), in which the affected siblings exhibited a 50% decrease in ADAM10 levels (species of 50 and 55 kDa), and decreased α-secretase activity (as determined by reduced levels of sAPPα) in CSF [24].

Other genetic studies have examined the presence of certain single nucleotide polymorphisms (SNPs), mostly in the *ADAM10* promoter region. Three studies conducted in Chinese populations [25,26,27] did not find differences in the distribution of genotypes between AD and controls, although there were some differences when stratified by age at onset [27] or *APOE*ε4 alleles [25]. However, the study of Bekris et al. [28] supported that a promoter haplotype, influenced by brain region and cell type, modulated ADAM10 expression and AD plaque scores.

Overall, all these studies provide strong evidence for the role of ADAM10 in the development of AD pathology, but clinical–genetic and biomarker correlations are not yet clearly established. In this study, we examine the hypothesis that certain *ADAM10* variants could increase the risk of AD by conferring earlier age-related downregulation of α-secretase, in opposition with other variants capable of maintaining high levels/activity of α-secretase at advanced ages. We also analyze the potential association of rare variants with reduced levels of ADAM10 in CSF.

## 2. Results

The population studied is summarized in Table 1.

### 2.1. Sequencing of ADAM10

The distribution of common variants was similar in AD cases and nonagenarians. Rare variants (MAF < 0.01) were found in 16 AD cases (15.5%, 11 different variants, one case carried three variants) and in 12 nonagenarians (12.5%, five different variants, six cases carried the same variant); chi-squared test *p* = 0.68 (Table 2). Several of the variants were group-specific and two of them found in AD cases were novel variants not reported in genome databases. However, only one of the variants was exonic: the Q170H variant, actually one of the described AD risk variants. This variant is reported with MAF = 0.0011 in the gnomAD database, and in our series was found in three AD cases (0.03) and one nonagenarian (0.01) (Fisher exact test *p* > 0.05).

All the other variants in AD cases and nonagenarians were intronic or located in the 3′ UTR region. These were present in single individuals or a couple of cases; an exception was SNP rs74016945 (3′UTR), which has a MAF close to 0.01 and was found in 6% of the nonagenarians (OR 0.146, Fisher exact test *p* = 0.057). It is remarkable that the AD cases with early-onset disease (<65 years *n* = 39) displayed more rare variants than late-onset cases (23% versus 8%, chi-squared test *p* = 0.058).

### 2.2. ADAM10 CSF Level Determinations by ELISA

We used three ELISA kits to accommodate all the CSF samples. Table 3 presents the ADAM10 levels in AD cases and controls per each kit. The range was quite different in the first kit versus the second and third (which belonged to the same batch and were more similar) and as a result could not be analyzed together.

Overall, ADAM10 levels had a short range in most samples. There were no differences in the first and second kit between cases and controls. There was a significant difference in the third kit, towards higher mean levels in the AD group (*p* = 0.006). However, it is noteworthy that the controls in this third kit were younger (50 ± 15 years, mean ± SD) than the controls in the previous kits (means of 67 and 61 years, Table 1).

**Figure 1:**
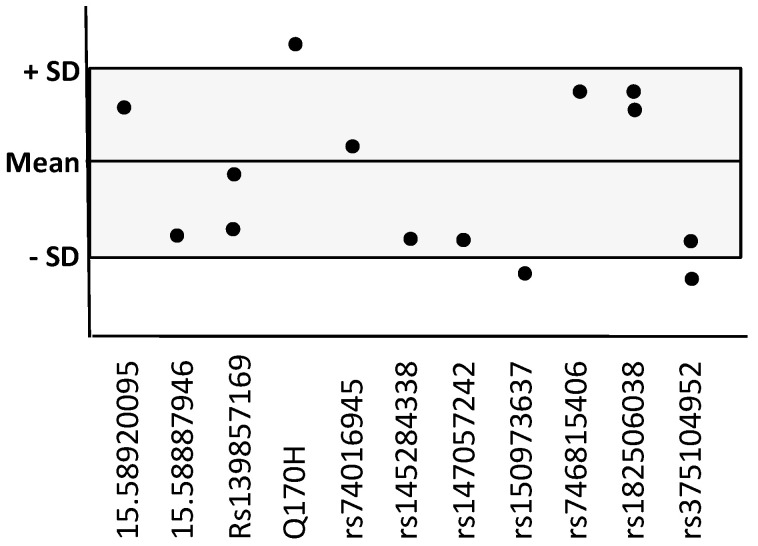
ELISA ADAM10 levels for each one of the 14 variant carriers, relative to the mean-SD of the AD non-carriers in the same kit (gray frame).

Comparisons of ADAM10 levels between early-onset versus late-onset AD cases (Table 3) produced significantly different results in samples from the first kit: late-onset cases (2.0 ± 0.3 ng/mL, mean ± SD) had lower levels than the early-onset group (2.3 ± 0.2 ng/mL, *p* = 0.012). There were no differences according to age at onset in AD cases included in the second and third kit. The 14 AD cases carrying rare variants did not show lower levels of ADAM10 in CSF compared to the non-carriers included in the same kit. The individual data for variant carriers (relative to the mean-SD of the AD non-carriers in the same kit) are represented in Figure 1. We did not analyze the CSF of two out of the three cases carrying the Q170H variant due to an absence of stored CSF samples. The Q170H variant carrier analyzed showed ADAM10 levels of 6.9 ng/mL in a kit (2nd) with mean ± SD= 6.4 ± 0.4 ng/mL.

ADAM10 levels were not different according to sex, except in the third kit, where men had higher levels than women (7.7 ± 0.6 ng/mL versus 7.2 ± 0.6, *p* = 0.034). There were no significant differences in ADAM10 levels according to *APOE* genotype. Finally, we did not find significant correlations between Aß42/40 ratios and ADAM10 levels (1stkit: Pearson correlation coefficient r = 0.21, *p* = 0.31; 2nd kit: r = 0.39, *p* = 0.055; 3rdkit: r = −0.01, *p* = 0.957).

### 2.3. ADAM10 CSF Species by Western Blot

**Figure 2:**
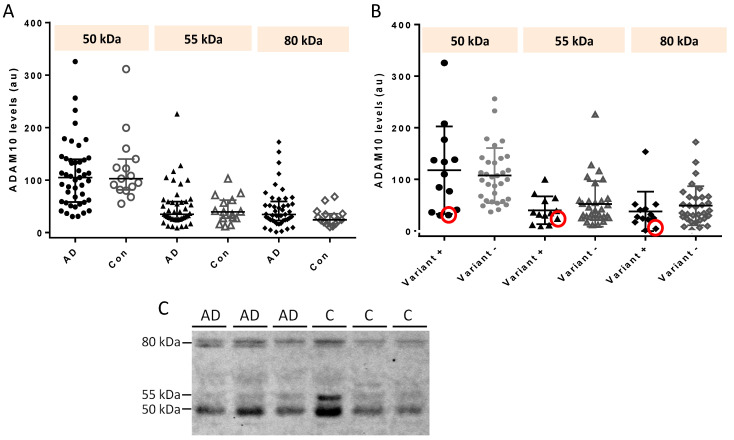
Densitometric quantification by Western blot of 50-kDa, 55-kDa, and 80-kDa ADAM10 bands (arbitrary units, au). (**A**) Comparison between AD cases and controls. (**B**) Comparison between AD cases carrying any rare genetic variants (variant+) versus non-carriers (variant−). Red circles point to values from the Q170H variant carrier. Bars are medians ± quartiles. Comparisons are not significant (Mann–Whitney U test). (**C**) Western blot images of three AD cases and three controls.

All the 45 AD samples (which included 13 AD carriers of rare variants) and the 15 controls analyzed by Western blot revealed the three ADAM10 immunoreactive species of 50 kDa, 55 kDa, and 80 kDa. Table 4 and Figure 2 summarize the data.

There were no significant differences in any of the species comparing AD cases and controls. We also did not find any differences when we compared the species of AD early-onset *versus* late-onset cases. The 13 AD cases carrying rare variants did not show lower levels of particular ADAM10 forms when compared with the 32 AD non-carriers. However, the AD carrier of the Q170H variant had substantially lower levels of all species (Table 4).

**Figure 3:**
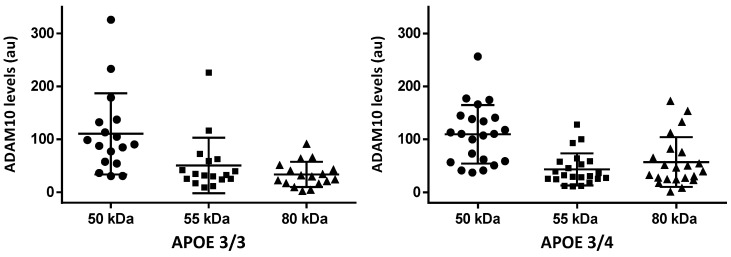
ADAM10 species in CSF samples of AD cases by Western blot (au: arbitrary units) stratified by *APOE* genotype (APOE 4/4 cases are not represented because they were only 4). Bars are medians ± quartiles. Comparisons are not significant (Kruskal–Wallis test).

There were no significant differences when comparing species in the AD group stratified by APOE genotypes. Figure 3 shows the data for *APOE*ε3/3 and *APOE*ε3/4 cases (cases with 4/4 were only *n* = 4). Finally, neither in the AD nor in the control group were significant correlations found between ADAM10 species and total ADAM10 levels as quantified by ELISA (Spearman correlation coefficients).

## 3. Discussion

In this study, we analyzed *ADAM10* variants in AD cases in relation to ADAM10 protein levels in CSF to test the hypothesis that certain rare variants could be associated with the age-related downregulation of α-secretase. Rare variants were found in up to 15% of the AD cases, but all except one were intronic and we did not find a consistent correlation of the variants with decreased protein levels.

The rationale for the study draws from previous reports emphasizing the crucial role of ADAM10 in non-amyloidogenic processing of APP, and as an attempt to enlighten genetic–biomarker correlations that are currently ambiguous. The strengths of our study include CSF analyses of a much larger sample size, a well characterized AD group (through positive CSF biomarkers) enriched in familial cases (82%), plus the nonagenarian population used for genetic comparison, the latter a unique representation of a genetic profile of brain aging resilience. Additionally, we sequenced the whole coding exons and flanking intronic regions of the gene, instead of mapping certain SNPs as has been performed3 in most previous studies. Furthermore, we conducted CSF ADAM10 analysis by means of two techniques: ELISA, more reproducible for quantitative purposes, and Western blot, which allows the separation and quantification of individual species.

Our genetic data showed a similar frequency of rare variants in AD cases and in controls (12 to 15%). We found a higher frequency of the Q170H variant, the only exonic change, among the AD cases (3%) as compared to nonagenarians (1%), but it did not reach significance. Although our AD series was enriched in familial cases (which could have been more prone to reveal at-risk genetic variants), our results are in line with two studies reporting no association between *ADAM10* variants and the risk of AD. Cai et al. [29] did not find the Q170H, R181G variants in a series of senile sporadic AD cases. Laws et al. [30] mapped 27 SNPs across the gene and did not identify particular *ADAM10* polymorphisms in AD cases as compared to a control population. Recent exome-wide studies, however, suggests a signal in *ADAM10* associated with AD risk [31,32], though prioritized variants in this gene are extremely scarce and occur only rarely. In fact, the *ADAM10* gene shows no tolerance whatsoever to loss of function mutations (observed/expected ratio 0.002, gnomAD browser). When a gene has a low o/e value (suggested threshold is < 0.35), it is under stronger selection for that class of variation than a gene with a higher value.

We also found some intronic variants that were specific to the AD group, including two in the 3′UTR region, but of unknown significance. These variants were present in 1–2% of the cases, as compared to global population frequencies of 0.008 to 0.00003% in gnomAD. Larger studies could clarify in the future whether any of these variants could increase the risk of developing AD. On the other hand, we found the SNP rs74016945 (located 3′UTR) in 6% of the nonagenarians while in population databases and AD was present in 0.9%. This difference nearly reached statistical significance, indicating that this variant could exert a certain influence in decreasing the risk for developing AD.

CSF ADAM10 protein levels, as quantified by a commercial ELISA kit specific for CSF samples, showed highly different ranges among kits. This prevented us from analyzing the three procedures together and decreased the power of the statistical analysis. Within the same kit, ADAM10 levels were quite homogeneous in cases and controls. We found significant single differences in some of the kits comparing AD and controls (toward higher levels in the AD group), with male patients exhibiting higher levels than females, or early-onset AD cases showing higher levels than late-onset patients. Increased CSF and plasma levels in mild AD cases has been reported by Pereira Vatanabe et al. [19], and a prospective study suggests that higher baseline plasma levels are predictive of cognitive decline [33]. The higher ADAM10 levels found in men could be meaningful considering AD is more prevalent among women; this result could be interpreted as suggestive of a protective biochemical phenotype in males. Further, higher levels in early- versus late-onset AD may suggest that late-onset disease is more closely related to the age-related downregulation of α-secretase, while early-onset AD would have other miscellaneous genetic causes involved. However, all these differences were not reproducible across all kits. More importantly, the AD variant carriers did not show lower levels than the non-carriers. In particular, the case with the Q170H variant had protein levels similar to AD non-variant carriers and similar to controls. Therefore, we did not find ADAM10 levels through this kit to be a clinical reliable biomarker.

Because we did not find much variability in the total ADAM10 levels through ELISA procedures, we extended the study of half of the samples (including the majority with rare variants) by conducting Western blot analysis. In prior research, this technique revealed a 50% decrease of mature ADAM10 species in the CSF of two siblings who were carriers of the nonsense p.Tyr167* *ADAM10* mutation [24]. In fact, we conducted the present study searching for genetic–CSF protein correlations in a larger series of familial AD cases because we previously found similarities in decreased protein CSF levels of the *ADAM10* mutation carriers and a small group of sporadic AD cases [24]. Sogorb-Esteve et al. [15] had also reported that the 50 and 55 kDa ADAM10 species were significantly decreased in sporadic AD cases compared to controls. However, in this larger set of AD cases, with a majority of familial cases, we did not find consistent differences with the controls. Nonetheless, it is noteworthy that the Q170H variant carrier had substantially lower levels of all species, although it is not possible to draw conclusions based on a single case.

There are some limitations to this study. The lack of rare exonic variants found in the AD group prevented any genetic–biomarker correlations. Intronic variants could exert an impact on the gene’s function, particularly those in promoter regions, but this remains a mere speculation. Additionally, the mean age of the AD cases studied is around 68 years. AD cases over the age of 80 years could have been more representative of the age-related downregulation of ADAM10 (even in the absence of rare genetic variants), though patients in this older age range do not undergo lumbar tap for AD core biomarkers as often as patients with younger onset dementia. In any case, we did not find significantly lower ADAM10 levels in the older patients or patients with older age at onset. Finally, we used a CSF-specific ADAM10 kit without previous data on its clinical or research performance. ADAM10 levels observed were within the detection range, but the different ranges obtained across kits prevented from analyzing the procedures together.

In summary, our study shows only intronic genetic variability in the *ADAM10* gene and quite homogeneous CSF protein levels in AD cases and controls. These data do not support the hypothesis of genetically determined downregulation of ADAM10 associated with the development of AD. Furthermore, protein levels in the CSF, as analyzed in this study, do not seem to be a reliable clinical biomarker for AD.

## 4. Methods

### 4.1. Participants

The population studied is summarized in Table 1. The main study group included 103 individuals with dementia of the Alzheimer type (DAT) recruited from the memory clinic of Fundación Jiménez Díaz (Madrid, Spain), a majority of them (82%) with a family history of dementia. The cases were selected from the DAT cohort because they had undergone a lumbar puncture for the study of core AD biomarkers in CSF with positive results. Family history was assigned a Goldman score [34], where 1 represents an autosomal dominant pattern, 2 familial aggregation of three or more, 3 one first-degree relative with dementia of onset <65 years, 3.5 one first-degree relative with dementia of onset over 65, and 4 no or unknown family history. In 83 of the 103 AD cases, there were CSF samples stored in which to analyze ADAM10 protein levels.

The controls for the genetic study were a group of 96 cognitively preserved nonagenarians (age range 90 to 105 years old). Cognitive status was confirmed by an interview with these individuals and their significant others, an MMSE over 28/30, and a brief neuropsychological assessment. All nonagenarians included were perfectly time-oriented, well aware of current events, and were independent for daily living activities. We chose to compare ADAM10 sequencing results between AD cases and cognitively preserved nonagenarians (as examples of resilience to AD) so as to analyze two groups that should be genetically further apart than AD cases and age-matched controls (who could develop AD a few years later). Furthermore, ADAM10 genotyping data of an extensive control population are available on databases (e.g., gnomAD). With this selection of groups (including a majority of familial AD cases), we expected to maximize the difference in genetic patterns, finding potentially rare ADAM10 variants increasing versus decreasing the risk for AD. These nonagenarians did not undergo lumbar puncture; therefore, for ADAM10 levels in CSF, we used a group of patients with no neurological diseases and cases with non-AD dementia (with negative CSF core AD biomarkers) as controls (described in Table 1). The same controls were used for the ELISA and Western blot analyses.

The study was approved by the Research Ethics Committee at the Fundación Jiménez Díaz and fulfilled current guidelines for ethical research according to the Helsinki Declaration. Informed consent for genetic and biomarker studies was provided by the patients or their surrogates.

### 4.2. Genetic Study

DNA was extracted from peripheral blood leukocytes using the QIAamp DNA blood Mini-kit (Qiagen). Sequencing of the *ADAM10* variants was conducted by Secugen S.L. using Next Generation Sequencing (NGS). Whole coding exons and flanking intronic regions were amplified by Ampliseq technology and sequenced using Miseq equipment (llumina), yielding a coverage of over 200x. Variants were filtered according to their frequency in genomic population databases (dbSNP, *Genome* Aggregation Database, and Exome Variant Server). Variants were considered rare when MAF < 0.01%. The potential impact of the variants was analyzed through Combined Annotation Dependent Depletion (CADD) scores (http://cadd.gs.washington.edu/score) [35] and the American College of Medical Genetics and Genomics recommendations [36]. We compared the percentage of rare variants between AD cases and nonagenarians using the chi-squared test. AD cases were stratified considering *APOE* genotype.

### 4.3. CSF Analyses

CSF samples from cases and controls had been stored at –80 °C. We conducted three types of analyses in CSF. First, we analyzed the core AD biomarkers Aβ42, Aβ40, total tau (t-tau), and phosphorylated tau (p-tau) levels using the Lumipulse G600II chemiluminescent immunoassay (Fujirebio Iberia, Barcelona, Spain), following the standardized commercial protocol. The cut-off points used to distinguish values consistent with AD were Aβ42 < 770 pg/mL, t-tau > 440 pg/mL, p-tau181 > 58 pg/mL, and Aβ42/40 ratio < 0.068. AD cases included in this study had biomarkers consistent with an A + T + N+ pattern [37].

Second, we analyzed ADAM10 levels by ELISA using a commercial kit specific for CSF samples (Human A Disintegrin and Metalloprotease 10 ELISA KitMBS037740_CSF, MyBioSource, Inc., San Diego, CA, USA), in accordance with the manufacturer-established protocol. Each kit allows for the study of 41 samples in duplicate and the detection range is 0.65 to 20 ng/mL. We compared the ADAM10 levels between AD cases and controls, between AD gene variant carriers and non-carriers, and between early- and late-onset AD cases by Student *t* test.

Third, a subset of AD samples and controls was further analyzed by Western blotting as previously described [15], to distinguish the three ADAM10 species present in human CSF: a truncated fragment lacking the intracellular C-terminal domain (sADAM10; ~50 kDa), a mature unprocessed full-length form (ADAM10f; ~55 kDa), and an immature full-length form retaining the prodomain (proADAM10; ~80 kDa) [16]. In brief, 25 µL of CSF samples were denatured at 98 °C for 7 min and resolved on 10% SDS-polyacrylamide slab gels under reducing conditions. Following electrophoresis, proteins were blotted onto nitrocellulose membranes (0.2 µm, Bio-Rad Laboratories GmbH, Munich, Germany), blocked with commercial Intercept^®^ PBS blocking buffer, and probed (overnight, 4 °C) with an anti-ADAM10 antibody specific for the mid-region, and as such was common to full-length and cleaved species (1:1000 diluted in PBS buffer, rabbit polyclonal; OAGA02442, Aviva Systems Biology, San Diego, CA, USA). Blots were then incubated for one hour with an IRDye 800 CW goat anti-rabbit secondary antibody (1:10,000 diluted in PBS buffer, LI-COR Biosciences, Lincoln, NE, USA) and imaged on an Odyssey Clx Infrared Imaging System (LI-COR). Densitometric quantification of the signal from the three species was analyzed using LI-COR software (Image Studio v 5.2.5, Lincoln, Nebraska, USA). All samples were analyzed in duplicate. The immunoreactive ADAM10 signal for each band was normalized to the immunoreactivity of the corresponding band from a CSF sample (aliquots from the same sample), resolved in all blots.

### 4.4. Statistical Analysis

Qualitative variables were summarized by frequencies and percentages. Age and ADAM10 levels determined by the ELISA technique were summarized as mean values and standard deviation. ADAM10 levels assessed by Western blot were summarized as median values and quartiles, due to the skewed distribution of the data. Associations between qualitative variables were tested using the chi-squared test, or the Fisher’s exact test when the requirements for using chi-squared were not met. Comparisons of ADAM10 levels between groups were performed using the Student’s t test for ELISA values, and the Mann–Whitney U-test for Western blot values. Statistical analyses were performed using R version 4.1.2 (R: A language and environment for statistical computing. R Foundation for Statistical Computing, Vienna, Austria. URL https://www.R-project.org/).

## Figures and Tables

**Table 1:** Population studied.

ADAM10 Analysis	AD Cases	Controls
**Sequencing**	***n* = 103**	***n* = 96**
Gender M/F	45/58	34/62
Age (yrs)	68 ± 6 (57–80)	93.2 ± 3 (90–105)
Age at onset (AO)	66 ± 6 (52–80)	--
MMSE	22.4 ± 3.6	28.7 ± 1.2
APOE	3/3 (42%); 3/4 (45%); 4/4 (12%)	2/3 (11%); 3/3 (83%); 3/4 (6%)
Goldman #	1 (9%), 2 (39%), 3 (9%), 3.5 (25%), 4 (18%)	--
**CSF ELISA**	***n* = 83**	*n* = 37 *
AD biomarkers	β42/β40 < 0.068, Ttau > 410, p-tau > 59	β42/β40 >0.068, Ttau <410, p-tau <40
1st kit	*n* = 27. AO: 66 ± 5; M/F: 11/16	*n* = 14. Age: 66 ± 5; M/F: 5/9
2nd kit	*n* = 27. AO: 63 ± 6; M/F: 13/14	*n* = 11. Age: 61 ± 10; M/F: 6/5
3rd kit	*n* = 29. AO: 68 ± 6; M/F: 15/14	*n* = 12. Age: 51 ± 16; M/F: 6/6
**CSF Western blot**	***n* = 45**	***n* = 15**
	AO: 66 ± 5 (52–75); M/F: 20/25	Age: 62 ± 12 (32–76); M/F: 8/7

AD cases belong to the same group through the three analyses (all underwent sequencing of ADAM10). Controls are the same for CSF biomarkers (ELISA and Western) but an independent group (nonagenarians) for genetic analysis. Age and MMSE data are mean ± SD (range). AO: age at onset. M: male/F: female. Ttau and p-tau in ng/mL. # Goldman scores for family history [29]. * Two controls are repeated in kit 2 and kit 3.

**Table 2:** Rare variants found in AD cases and preserved nonagenarians.

*ADAM10* Variants dbSNP ID	MAF gnomAD (v3.1.1)	Present in *n* AD Cases/Frequency	Present in *n* Nonagenarians/Frequency
**Non-described (chr pos)**			
15.58920095 (A-G)	--	*n* = 1 0.01	--
15.58887946 (3′UTR C-T)	--	*n* = 1 0.01	--
**Rare variants**			
rs139857169	0.007	*n* = 2 0.02	*n* = 2 0.02
rs61751103 (Q170H)	0.0014	*n* = 3 0.03	*n* = 1 0.01
rs74016945 (3′UTR)	0.0089	*n* = 1 0.01	*n* = 6 0.06 *
rs192779774 (G-T)	0.0042	--	*n* = 2 0.02
rs201948093	0.0018	--	*n* = 1 0.01
rs145284338	0.0068	*n* = 1 0.01	--
rs147057242(3′UTR)	0.0052	*n* = 1 0.01	--
rs150973637 (A-C)	0.00013	*n* = 2 0.02	--
rs746815406	0.00003	*n* = 1 0.01	--
rs182506038(3′UTR)	0.00054	*n* = 2 0.02	--
rs375104952 (T-C)	0.00010	*n* = 2 0.02	--

* Only comparison close to significance: OR 0.147, Fisher exact test 0.055; MAF: minor allele frequency. Gnom AD: *Genome* Aggregation Database.

**Table 3:** CSF ADAM10 analyzed by ELISA.

ADAM10 (ng/mL)	AD Cases n = 83	Controls n = 37	*p* (Student Test)
**1 kit**	Total n = 27 2.1 ± 0.3 Early-onset (n = 9) 2.3 ± 0.2 Late-onset (n = 18) 2.0 ± 0.3 Variant + (n = 4) 2.0 ± 0.3	2.2 ± 0.1 (n = 14)	**0.59** Early vs late-onset: 0.012 Variant + vs-: ns
**2 kit**	Total n = 27 6.4 ± 0.4 Early-onset (n = 15) 6.4 ± 0.4 Late-onset (n =12) 6.3 ± 0.4 Variant + (n = 8) 6.4 ± 0.4	5.8 ± 1.1 (n = 11)	**0.16** Early vs late-onset: 0.86 Variant + vs-: ns
**3 kit**	Total n = 29 7.5 ± 0.6 Early-onset (n = 8) 7.4 ± 0.6 Late-onset (n = 21) 7.5 ± 0.6 Variant + (n = 2) 7.7 ± 0.1	6.7 ± 0.9 (n = 12)	**0.006** Early vs late-onset: 0.79 Variant + vs-: ns

Data are mean ± SD. ns = not significant. Variant + (carriers of rare variants), variant–non-carriers.

**Table 4:** ADAM10 species in CSF analyzed by Western blot.

	50 kDa	55 kDa	80 KDa
**AD cases (*n* = 45)**	105 (58.8, 138)	34.7 (25.4, 58.7)	34.4 (23.4, 54.4)
**Controls (*n* = 15)**	103 (84.3, 132)	39.3 (27.5, 59.2)	24.4 (18.1, 35.1)
	*p* = 0.567	*p* = 0.986	*p* = 0.140
**Early-onset AD (*n* = 16)**	113 (64.8, 139)	38.0 (26.2, 58.8)	27.4 (16.8, 57.7)
**Late-onset AD (*n* = 29)**	98.7 (58.8, 137)	32.2 (25.4, 57.2)	39.6 (25.0, 54.4)
	*p* = 0.749	*p* = 0.953	*p* = 0.245
**AD variant carriers (*n* = 13)**	110 (41.1, 138)	34.7 (24.0, 58.7)	27.3 (22.5, 43.0)
**AD non-carriers (*n* = 32)**	102 (61.1, 135)	35.9 (25.5, 58.1)	37.3 (24.1, 65.5)
	*p* = 0.940	*p* = 0.582	*p* = 0.225
**Q170H carrier (*n* = 1)**	30.8	24.0	4.6

Data are medians (quartiles). *p* values according to Mann–Whitney U test.

## Data Availability

Sequencing data from this study is available at Secugen S.L., upon request. Data on CSF analysis is available at the Neurology Department (Fundación Jiménez Díaz), upon request.
